# Recent Technical Developments in ASL: A Review of the State of the Art

**DOI:** 10.1002/mrm.29381

**Published:** 2022-08-19

**Authors:** Luis Hernandez‐Garcia, Verónica Aramendía‐Vidaurreta, Divya S. Bolar, Weiying Dai, Maria A. Fernández‐Seara, Jia Guo, Ananth J. Madhuranthakam, Henk Mutsaerts, Jan Petr, Qin Qin, Jonas Schollenberger, Yuriko Suzuki, Manuel Taso, David L. Thomas, Matthias J. P. van Osch, Joseph Woods, Moss Y. Zhao, Lirong Yan, Ze Wang, Li Zhao, Thomas W. Okell

**Affiliations:** ^1^ FMRI Laboratory University of Michigan Ann Arbor Michigan USA; ^2^ Department of Radiology Clínica Universidad de Navarra Pamplona Spain; ^3^ Center for Functional Magnetic Resonance Imaging, Department of Radiology University of California at San Diego San Diego California USA; ^4^ Department of Computer Science State University of New York at Binghamton Binghamton NY USA; ^5^ Department of Bioengineering University of California Riverside Riverside California USA; ^6^ Department of Radiology UT Southwestern Medical Center Dallas Texas USA; ^7^ Department of Radiology & Nuclear Medicine Amsterdam University Medical Center, Amsterdam Neuroscience Amsterdam The Netherlands; ^8^ Helmholtz‐Zentrum Dresden‐Rossendorf Institute of Radiopharmaceutical Cancer Research Dresden Germany; ^9^ The Russell H. Morgan Department of Radiology and Radiological Science Johns Hopkins University Baltimore Maryland USA; ^10^ Wellcome Centre for Integrative Neuroimaging, FMRIB, Nuffield Department of Clinical Neurosciences University of Oxford Oxford United Kingdom; ^11^ Division of MRI research, Radiology Beth Israel Deaconess Medical Center and Harvard Medical School Boston Massachusetts USA; ^12^ Department of Brain Repair and Rehabilitation UCL Queen Square Institute of Neurology London United Kingdom; ^13^ C.J. Gorter Center for high field MRI, Department of Radiology Leiden University Medical Center Leiden The Netherlands; ^14^ Department of Radiology University of California La Jolla California USA; ^15^ Department of Radiology Stanford University Stanford California USA; ^16^ Department of Radiology, Feinberg School of Medicine Northwestern University Chicago Illinois USA; ^17^ Department of Diagnostic Radiology and Nuclear Medicine University of Maryland School of Medicine Baltimore Maryland USA; ^18^ Key Laboratory for Biomedical Engineering of Ministry of Education, College of Biomedical Engineering & Instrument Science Zhejiang University Zhejiang People's Republic of China

**Keywords:** arterial spin labeling, CBF, MR imaging, perfusion, technical advances, vascular imaging

## Abstract

This review article provides an overview of a range of recent technical developments in advanced arterial spin labeling (ASL) methods that have been developed or adopted by the community since the publication of a previous ASL consensus paper by Alsop et al. It is part of a series of review/recommendation papers from the International Society for Magnetic Resonance in Medicine Perfusion Study Group. Here, we focus on advancements in readouts and trajectories, image reconstruction, noise reduction, partial volume correction, quantification of nonperfusion parameters, fMRI, fingerprinting, vessel selective ASL, angiography, deep learning, and ultrahigh field ASL. We aim to provide a high level of understanding of these new approaches and some guidance for their implementation, with the goal of facilitating the adoption of such advances by research groups and by MRI vendors. Topics outside the scope of this article that are reviewed at length in separate articles include velocity selective ASL, multiple‐timepoint ASL, body ASL, and clinical ASL recommendations.

## Abstract 2

Click here for author‐reader discussions

## 1 INTRODUCTION

Since its introduction in the early 1990s, arterial spin labeling (ASL) has proved to be a powerful noninvasive, noncontrast alternative to conventional perfusion imaging methods.^1^, ^2^ The publication of a consensus paper on the clinical implementation of ASL in 2015^3^ was instrumental in the adoption of ASL brain imaging in the clinic and provided a common reference for researchers. Also, it provided expert guidelines for ASL sequence implementation for the major MR manufacturers, who now all offer the same labeling strategy (pseudo‐continuous ASL [PCASL]) and similar readouts (3D spiral or gradient and spin echo [GRASE]). Consequently, clinical applications of ASL have significantly increased, and a benchmark for comparison of future developments was established.

**FIGURE 1:**
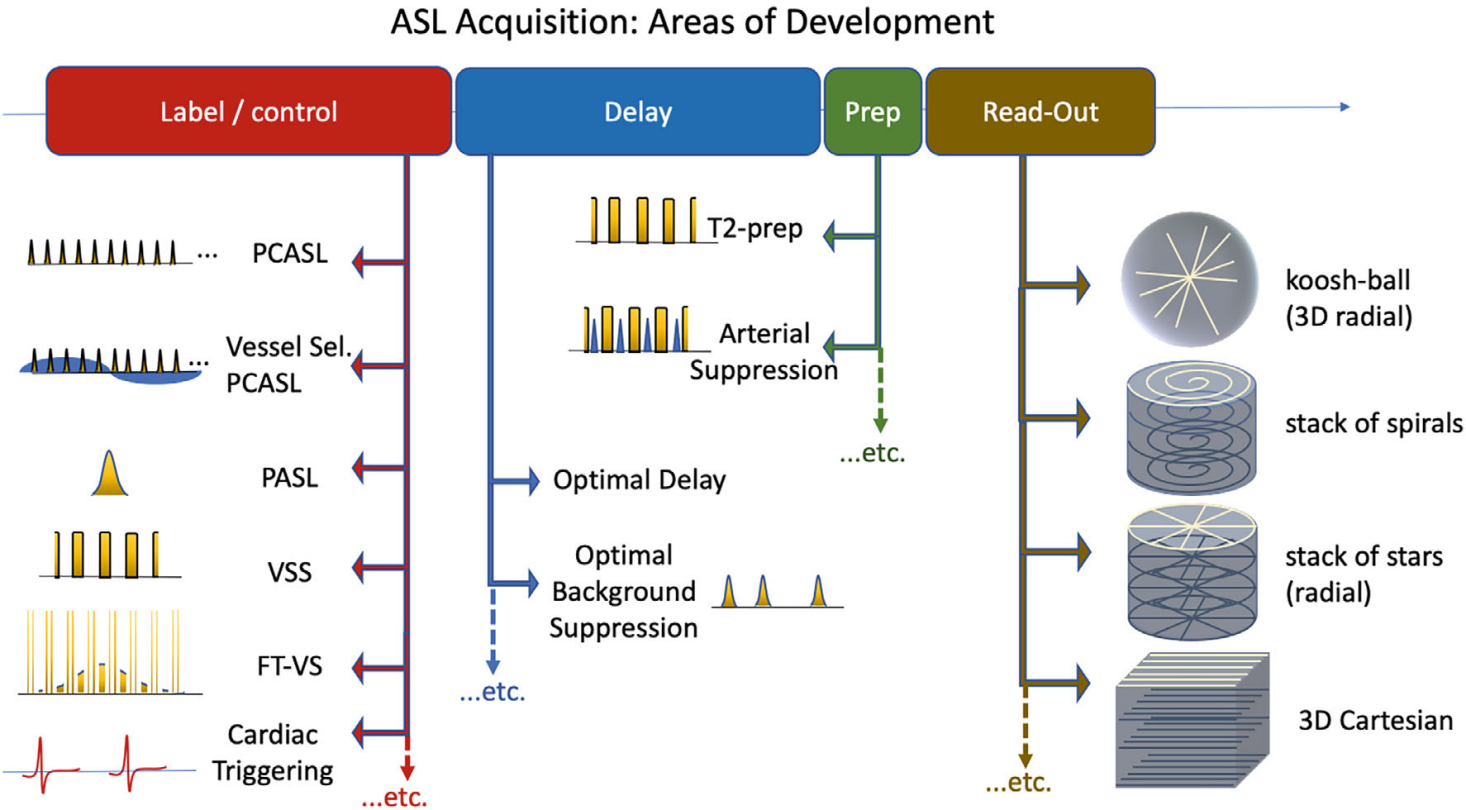
The above diagram depicts the typical components of ASL pulse sequences, highlighting some advancements that have been made in recent years.

Nevertheless, new variants and improvements in ASL acquisition design (see Figure 1) and ancillary measurements have been developed since 2015, aiming to improve image quality, provide more accurate cerebral blood flow (CBF) quantification or measure additional physiological parameters, and extend applications of ASL beyond the brain.

This paper will review new capabilities of ASL including vessel selective ASL, quantification of parameters beyond perfusion, the use of fingerprinting and deep learning (DL) techniques, ASL‐based fMRI, and postprocessing techniques to improve image quality. We will describe these new techniques to provide a high‐level intuition and some suggestions for their implementation, which are based on the experience of the authors, with endorsement by the perfusion study group of the International Society for Magnetic Resonance in Medicine (ISMRM). Our goal is to facilitate and promote the adoption of such advances by research groups and by MR scanner vendors.

Some topics will be out of the scope of this overview and will be reviewed in separate articles. For example, velocity selective ASL is one of the most significant innovations in the area of ASL as it eliminates arterial transit time confounds and can provide a significant boost in SNR. Also, quantitative ASL using multiple timepoints allows more accurate estimation of perfusion as well as additional parameters, particularly the arterial transit time. Furthermore, great advances have also been made in body ASL due to innovative technical developments. These topics are quite extensive; each merits a review article in itself and thus will not be covered in this article.

## 2 READOUT AND TRAJECTORIES

The consensus paper recommended 3D segmented imaging sequences with stack of spiral with fast spin echo (FSE) or Cartesian GRASE. 2D multi‐slice methods based on EPI or 2D spirals with or without simultaneous multi‐slice excitation are also possible^4^, ^5^, ^6^ and may be useful at high field strengths where power deposition limits prohibit the use of multiple refocusing pulses, but 3D methods tend to be advantageous in terms of SNR and the effectiveness of background suppression, allowing full brain coverage in acceptable scan times.

However, both GRASE and FSE readouts use long echo trains to encode all the slices in the volume. T_2_ decay along the echo train results in blurring in the slice direction, whereas T_2_* decay between refocusing pulses introduces in‐plane blurring. Blurring can be mitigated to some extent by splitting the readout into more segments, but at the cost of a longer time to acquire each volume (reducing the temporal resolution) and increased sensitivity to intershot motion. Recently, several technical developments have been proposed to overcome some of these issues using novel acquisition schemes and image reconstruction techniques.

### 2.1 Improvements in 3D segmented readouts

#### 2.1.1 Variable flip angle design

In conventional 3D readouts, the refocusing flip angle is constant and the resulting signal decays from one echo to the next, leading to through‐slice blurring. Variable flip angle designs can result in a more consistent signal across the echo train, reducing the signal modulation and thereby the blurring effect. For example, an extended phase graph approach can be used to design a flip angle schedule for 3D‐GRASE, greatly reducing the width of the blurring point spread function.^7^ This approach can also be combined with echo amplitude scaling of the k‐space data to target a specific signal response.^8^ In addition to improving image quality, this approach also offers the possibility of significantly reducing the power deposition arising from the FSE echo‐train.

#### 2.1.2 Improvements to spiral readouts

Spiral trajectories can be quite sensitive to poor magnetic field homogeneity, eddy currents, and imperfections in gradient performance, resulting in significant image blurring and distortion, especially when using high gradient slew‐rates.^9^ One solution is to measure the actual gradient trajectory and use this to improve image reconstruction.^10^, ^11^, ^12^ Another approach relies on improvements to spiral trajectories using a combination of 3D spiral in/out (referred to as cylindrical distributed spiral) to reduce signal dropout and image blurring when compared to standard stack‐of‐spirals.

#### 2.1.3 Accelerated 3D readouts

The TR of ASL is mainly limited by the labeling duration and postlabel delay since the data acquisition time is only a small fraction of the TR, so accelerated sampling schemes do not significantly reduce the TR. However, undersampled 3D trajectories have been explored to reduce the echo train duration and/or the required degree of segmentation, which can be leveraged to improve temporal resolution and robustness to motion as well as mitigating blurring artifacts.

In Cartesian imaging, for example, parallel imaging reconstruction using an improved GRAPPA kernel^13^ provided higher SNR and reduced blurring due to the shortened TE and readout times.^14^, ^15^ For non‐Cartesian sampling, 1D acceleration in the slice direction combined with variable‐density spirals can be used to reduce the echo train length, resulting in a significant reduction in blurring.^16^, ^17^

Controlled Aliasing In Parallel Imaging Results In Higher Acceleration (CAIPIRINHA) trajectories can further improve image quality for 3D‐GRASE by reducing g‐factor noise amplification.^18^ A time‐dependent CAIPIRINHA sampling pattern has additional advantages of allowing coil sensitivity maps to be generated from the different k‐space data acquired over time, as well as being better suited to more sophisticated reconstruction approaches using spatiotemporal regularization.^19^

### 2.2 Cartesian FSE

Segmented FSE acquisitions with Cartesian encoding, where 1 line of k‐space is acquired after each refocusing pulse, are workhorses of volumetric imaging, having excellent off‐resonance robustness and anatomical fidelity. Whereas this makes FSE particularly attractive for body ASL and high‐resolution ASL, long acquisition times are a major limiting factor if large volume coverage is required.

Using a reduced Field of view (FOV) with selective excitation allowed the benefits of volumetric Cartesian encoding for renal imaging to be demonstrated.^20^ More time‐efficient acquisitions with spiral re‐ordering on a Cartesian grid,^21^ variable‐density sampling combined with compressed sensing reconstruction for body,^22^ and brain imaging^23^ have also been demonstrated.

### 2.3 Radial trajectories

Whereas conventional trajectories (e.g., 2D/3D EPI or spirals) are very efficient at covering large amounts of k‐space quickly, they generally have a fixed spatial/temporal resolution and suffer from artifacts due to off‐resonance effects and motion between shots. Radial k‐space trajectories, which acquire a single line of k‐space at a time through its center with different orientation, sample fewer k‐space points but allow the retrospective choice of spatial and temporal resolution for reconstruction when using golden ratio sampling^24^, ^25^; are intrinsically robust to motion; do not suffer from significant distortion, blurring, or signal dropout artifacts; and tolerate relatively high levels of undersampling, particularly when combined with advanced reconstruction techniques (see below).

Radial trajectories have been used fairly extensively for ASL angiography (see below), and more recently for assessing the labeling efficiency of velocity‐selective ASL preparations^26^; however, a few new methods have also explored their use for ASL perfusion imaging. In the Combined Angiography and Perfusion using Radial Imaging and ASL (CAPRIA) approach,^27^ a PCASL preparation is followed by a continuous golden ratio readout. Dynamic angiographic images are reconstructed using a small number of radial spokes from early timepoints, while the labeled blood still resides within the arteries. This results in a high temporal resolution and a high undersampling factor, but the sparse nature and high SNR of the angiographic signal means good quality images can still be reconstructed. Using the same raw data, perfusion images can be reconstructed from later time points once the labeled blood arrives at the tissue.

A golden ratio readout can also be combined with a time‐encoded ASL preparation: this means fewer excitation pulses are needed to span a range of effective postlabeling delays,^28^ allowing higher flip angles to be used without causing excessive signal attenuation. This boosts the SNR, in addition to the noise‐averaging benefit of time‐encoding. Although potentially more time‐efficient than separately acquired angiography and perfusion imaging, further studies are required to refine these techniques and test them in clinical cohorts.

### 2.4 Cardiac triggering

The variability of blood flow velocity in the brain‐feeding arteries affects the ASL labeling efficiency (in CASL and PCASL) and arterial transit time. These effects have been tested with cardiac gating in pulsed,^29^, ^30^ pseudo‐continuous,^31^ and velocity/acceleration‐selective ASL.^32^ For example, shorter bolus arrival time and a 16% higher perfusion signal in gray matter (GM) were found when triggering a pulsed ASL (PASL) labeling module at systole compared to diastole, although the signal was similar at long TIs.^29^ Larger signal variations across the cardiac cycle have been demonstrated for velocity selective ASL (36%) and acceleration selective ASL (64%) compared to PCASL (25%).^32^ Similarly, stability gains were found in vessel‐selective ASL by triggering.^33^

A PCASL study^31^ triggered the end of the labeling period to a specific cardiac phase with a long labeling duration (> 7 s) and found no significant differences in vivo in the mean ASL signal and its stability. However, a second study^34^ tested a nontriggered‐ versus a cardiac‐triggered standard PCASL sequence with the parameters suggested in the consensus paper.^3^ The nontriggered PCASL sequence showed signal fluctuation near large vessels in single‐shot acquisitions and also more artifacts in segmented acquisitions, whereas the cardiac triggered sequence demonstrated higher temporal SNR.

Cardiac triggering improves stability at a cost of increased dead‐time in the sequence while waiting for the next cardiac trigger. Triggers should be applied to the start of labeling because triggering of the readout would lead to differences in postlabeling delay (PLD) between acquisitions and thus imperfect subtraction of static signal between label and control condition.

### 2.5 Suggestions

The use of moderately segmented 3D readout schemes continues to be recommended for ASL due to their high efficiency and SNR, as well as their ability to achieve spatially uniform background suppression. The use of parallel imaging with relatively low acceleration factors (e.g., 2 or 3) is also recommended when available (e.g., for Cartesian trajectories), particularly when combined with low g‐factor methods such as CAIPIRINHIA. We encourage the further development and validation of newer techniques before they are used for clinical research applications. At this time, there is not a sufficient amount of evidence to recommend the general use of cardiac triggering with ASL.

## 3 Advances in image reconstruction and processing

### 3.1 Advanced reconstruction techniques

ASL‐perfusion imaging has some inherent properties that make it well suited for acceleration and reconstruction using compressed sensing methods. Particularly, compressed sensing has been shown to perform well when applied to ASL difference images by leveraging sparsity across the averages^22^ or using a total generalized variation constraint in combination with a time‐dependent CAIPIRINHA sampling pattern.^35^ Multi‐delay ASL images can be further improved by additionally exploiting the redundancy among images (temporal sparsity) with different labeling duration and postlabel delays. For example, an over‐complete dictionary was built from the perfusion model and was used to sparsify the acquired ASL signal.^36^ This helped reject noise and motion artifacts that could not be described by the perfusion signal model.

### 3.2 Noise reduction

Many strategies have been developed to improve ASL SNR using image‐processing techniques. Spatial smoothing is a routine procedure for suppressing random noise in MRI and has been used frequently for ASL^14^; however, this further reduces the already low spatial resolution and blurs perfusion differences between tissue types. This can be partly addressed by using Wavelet denoising^37^ or by a spatial kernel as part of partial volume correction approaches.^38^, ^39^ High‐pass filtering can remove temporal noise^36^ as the perfusion signal encoded in the label‐control acquisition paradigm is located in the high‐frequency band.^40^, ^41^

**FIGURE 2:**
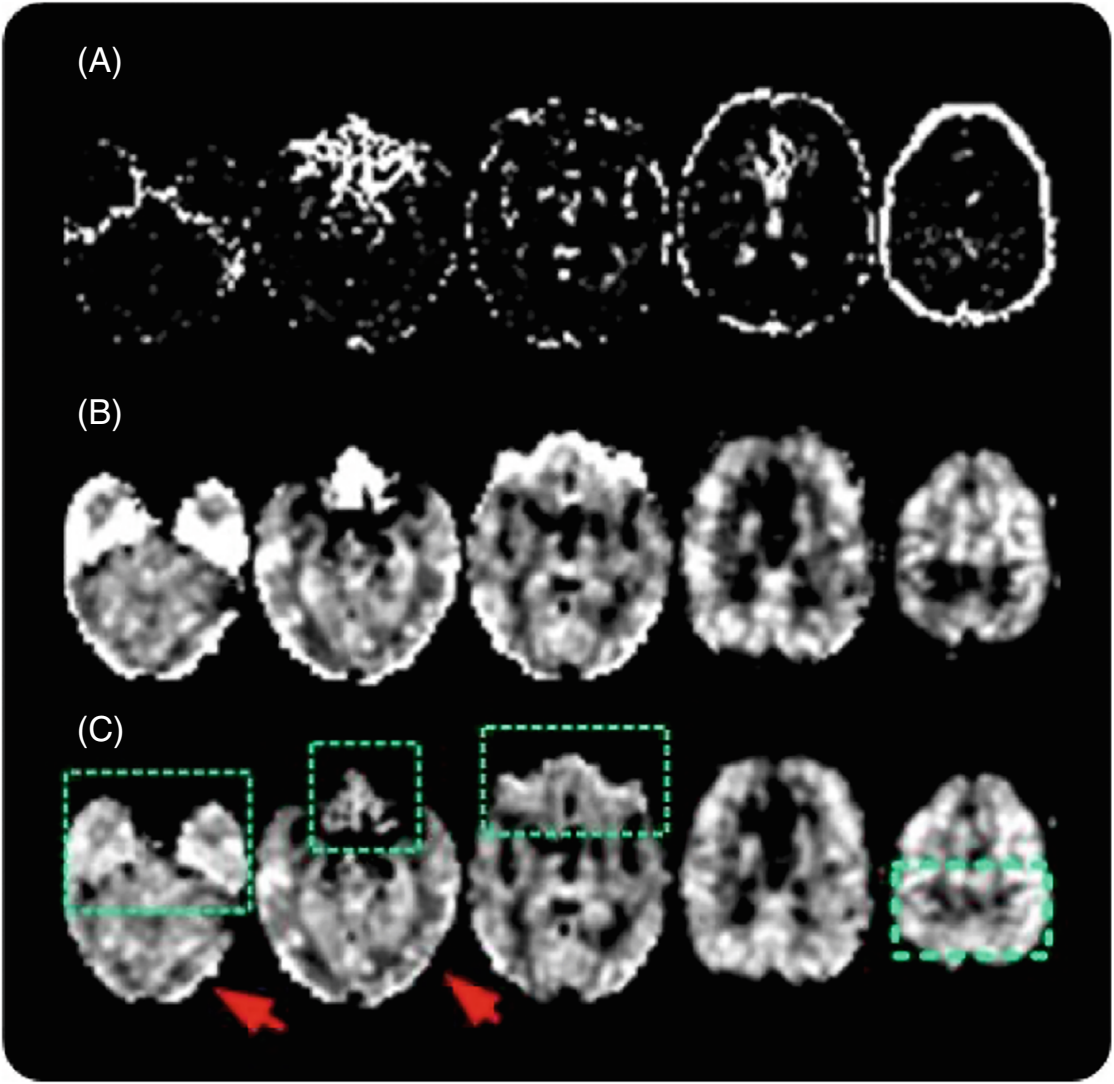
*ASL CBF images of a cocaine addicted patient processed (A) without outlier cleaning, (B) using the original adaptive outlier cleaning algorithm, (C) using the prior‐guided slicewise outlier cleaning algorithm. Outlier cleaning provided substantial CBF quality improvement in this case. Green boxes and red arrows were used to mark the places with significant CBF differences. (Figure reproduced from Ref*.^44^ *with permission from the author.)*

Outliers, caused by physiological fluctuations or subject motion, are a major challenge for ASL MRI, especially due to the limited number of samples.^39^ Robust fitting^42^ can address outliers at the voxel‐level, although it does not take spatial information into account. Several empirical algorithms were introduced to remove outlier volumes or slices before calculating the final CBF map, which can be identified based on motion parameters and variation in the CBF time series^39^ or using a M‐estimator.^43^ An adaptive outlier cleaning algorithm (see Figure 2) can iteratively identify outlier volumes based on the correlation of each remaining volume to the current mean image.^44^ This approach can be improved using structural information regularization,^45^ using a prior‐guided slice‐wise adaptive outlier cleaning method,^46^ or by accounting for relative motion.^47^

Alternatively, spatial priors can be used on the resulting CBF and/or arterial transit time (ATT) maps^48^, ^49^ to reduce the effect of outliers, or a total generalized variation regularized spatial–temporal filtering algorithm can be used for directly denoising the raw ASL images.^19^

**FIGURE 3:**
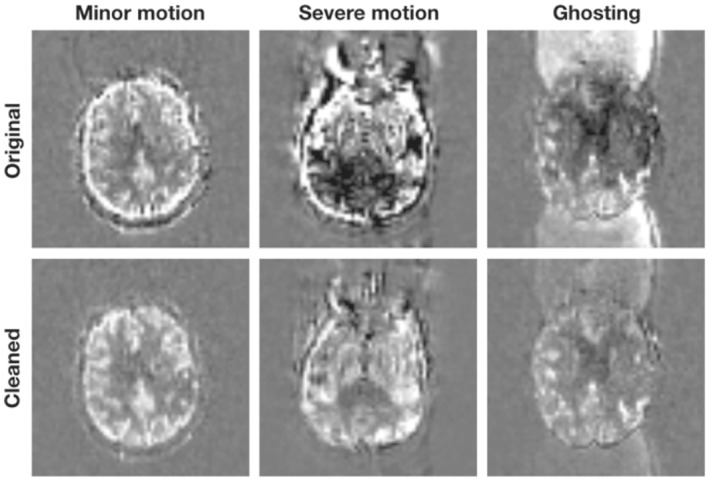
*Independent Component Analysis‐based denoising: some example data from the study by Carone* et al.^50^ *before (top row) and after (bottom row) denoising using FSL FIX. In this study of acute stroke patients, ASL data were acquired at 5 different PLDs in 4.5 min. Each image above shows the average subtraction image after motion correction at 1 PLD (6 label‐control pairs), where the effect of denoising is most apparent. This approach gives a considerable reduction in artifacts related to motion and other sources, such as ghosting. Data kindly provided by Davide Carone and the AMICI study team*.

Another strategy to denoise ASL data is to decompose the signal into components and then regress out the “noise” components. One approach is to use independent component analysis with manual or automatic classification of components (e.g., by assessing if the spatial/temporal variations match the expected perfusion signal), which results in improved SNR and repeatability^50^, ^51^, ^52^ (Figure 3). Similarly, the component‐based noise correction method extracts principal components from noise regions of no interest, which can be used as covariates in a general linear model and improve the stability of the perfusion signal.^53^, ^54^ Alternatively, a low‐rank and sparse decomposition can separate the ASL image series into slowly changing perfusion and spatially sparse noise component.^55^

Recently, DL has been utilized for simultaneous denoising and resolution improvement in ASL,^56^, ^57^, ^58^ and various approaches have allowed a significant acquisition time reduction without sacrificing CBF quantification quality.^59^, ^60^ Unsupervised DL ASL denoising algorithms using autoencoder networks have also been proposed,^61^ reducing the burden of generating large amounts of training data. Deep convolutional neural networks have been used to enhance image quality of multi‐timepoint ASL data acquired with a low number of averages,^59^ showing a 40% higher accuracy than the conventional averaging method when tested on ASL data of stroke patients.

Different ASL acquisition strategies introduce different noise patterns, making it necessary to fully evaluate the capability of a model to transfer from one type of ASL data or population to another. However, it is important to be careful not to “over‐denoise” functional ASL images, as sometimes the activation itself is correlated with components identified as noise, and suppressing too many temporal components may artificially increase functional connectivity.

### 3.3 Partial volume correction

**FIGURE 4:**
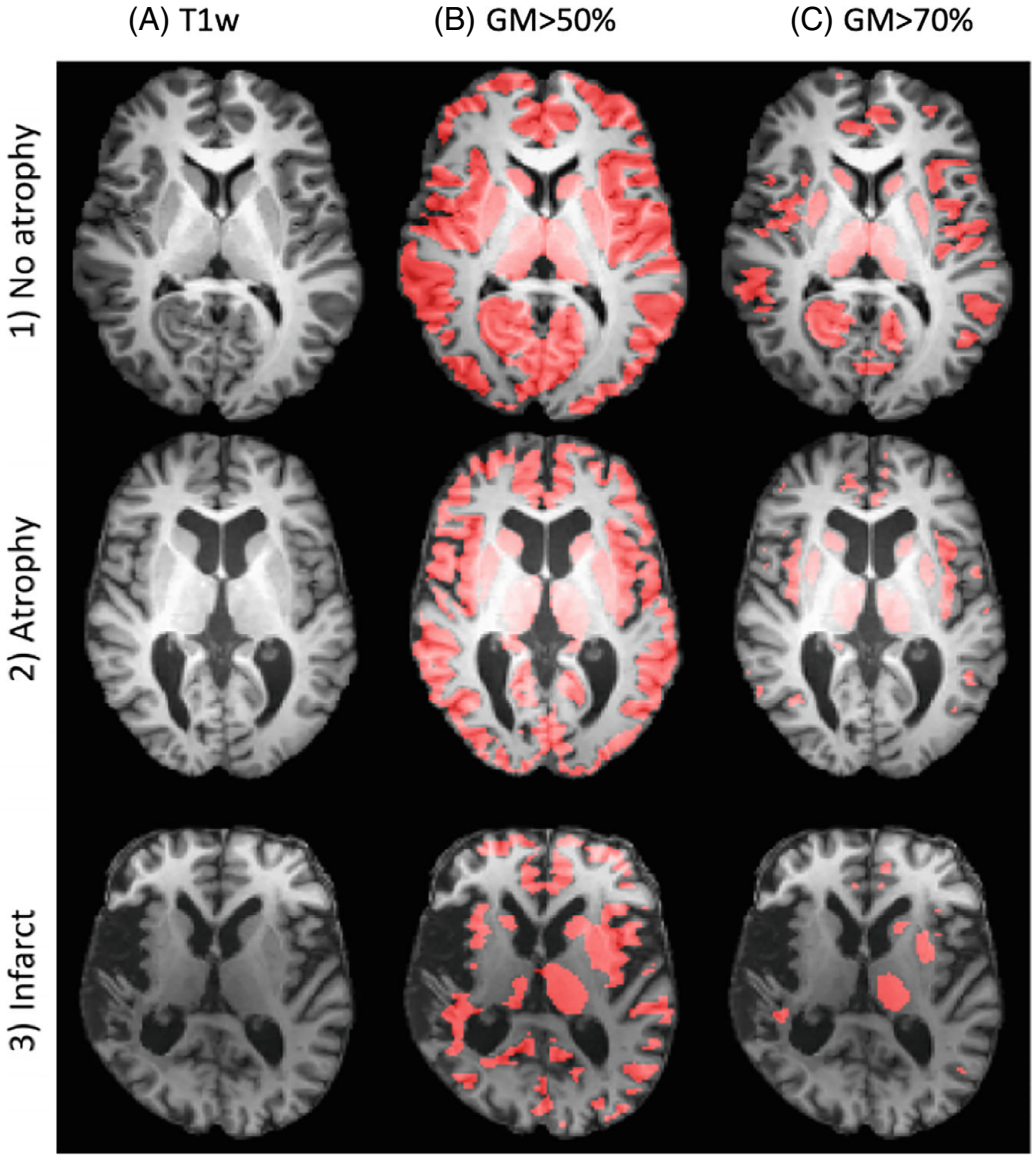
*Demonstration of the need for PVC in ASL using 3 subjects: (1) a healthy adult, (2) an older adult with atrophy, and (3) an older adult with a unilateral infarct. (A) Native space structural T*_*1*_ *‐weighted (T*_*1*_ *w) images. (B, C) T*_*1*_ *w images overlaid, in red, with the GM tissue segmentations. The GM segmentation was smoothed to the resolution of ASL images to express the partial volume of GM in each voxel of the ASL images. This GM image was then thresholded at b) 50% and c) 70% to create a mask of voxels with a GM content above the threshold. The 70% threshold on GM images is typically used for calculating the mean CBF in GM. These images show that, especially in clinical cases and thin cortical regions, only a fraction of ASL voxels contain sufficient GM to pass the thresholding for GM CBF calculation, thus introducing a spatial bias in the resulting mean GM CBF. Use of PVC to obtain corrected GM CBF values is thus recommended, and using this in conjunction with a 50% threshold GM mask for the calculation of mean GM CBF results in reasonable spatial coverage while minimizing PV effects (Figure reproduced from Ref*.^174^ *with permission from the authors)*. GM, gray matter; PVC, partial volume correction.

ASL spatial resolution is typically much lower than the cortical thickness (average value ∼2.5 mm vs. typical ASL resolutions of 4 × 4 × 4 mm^3^). In superficial brain regions, individual voxels are therefore highly likely to contain a mixture of GM, white matter (WM), and cerebrospinal fluid (CSF), which is known as the partial volume (PV) effect. Given that GM perfusion is approximately 2–5 times greater than WM perfusion^62^, ^63^ PV will have a large effect on CBF quantification. In ASL, the primary focus is often on GM‐CBF. PV effects bring 2 issues here: 1) actual GM content is still variable in nominally “GM voxels” causing potential GM‐CBF underestimation; and 2) the spatial distribution of predominantly GM voxels varies between subjects, causing a potential evaluation bias (Figure 4). The importance of PV effects grows in longitudinal and cross‐section studies where cortical thickness varies in time and across groups.^64^, ^65^ Several algorithms have been proposed to correct for PV‐effects at the voxel level using fractional GM and WM maps obtained from segmenting structural images. These algorithms either assume a locally homogeneous GM and WM CBF,^62^ leveraging the different kinetics in GM and WM (along with spatial regularization),^38^ or use GM volume as a covariate in the statistical analysis.^66^

The quality of the fractional GM and WM maps, along with coregistration, distortion correction, and resolution errors^67^ also propagate into the PV correction. However, these errors would have a similar influence on non‐PV corrected GM‐CBF evaluation using a GM mask,^68^ or alternative approaches using tissue classification from inversion recovery or a similar readout sequence.^69^ It is important to note that the partial volume effect is a methodological artifact. Correcting for it allows the investigator to examine changes in perfusion and GM volume as separate effects, even in patients where both are changing concurrently. For the latter, GM volume could be a covariate in statistical analyses; for the former, PV correction is more appropriate. These 2 issues are currently usually not separately addressed.

### 3.4 Suggestions

When processing ASL data, we recommend the use of motion correction (unless very strong background suppression is performed) and consideration of at least 1 denoising technique (such as adaptive outlier cleaning or component‐based methods) if there are sufficient measurements to support them. Partial volume correction as an additional analysis is strongly recommended for studies focusing on specific tissue types, such as GM, especially if a difference in tissue volumes, for example, due to atrophy, is expected between participants or cohorts.

## 4 OTHER PARAMETERS BEYOND PERFUSION

ASL data can also be used to quantify a number of other hemodynamic parameters, such as arterial transit time, arterial blood volume, arterial and venous blood oxygenation, and the metabolic rate of oxygen consumption.

### 4.1 Blood oxygenation and oxygen consumption

Spin labeling methods can be creatively applied to measure venous oxygen saturation (S_v_O_2_, or Y_v_ are commonly used in the literature), from which Oxygen Extraction Fraction (OEF) and cerebral metabolic rate of oxygen (CMRO2) can be subsequently estimated. All 3 parameters are important indicators of brain health and function and are often perturbed in states of disease.

One class of methods to estimate Y_v_ first measures the T_2_ of venous blood, which is then calibrated to Y_v_ using empirical or theoretical relationships because blood T_2_ is directly related to the blood oxygenation fraction.^70^ OEF can then be estimated using the derived venous oxygenation (Y_v_) along with a measured or assumed value of arterial oxygenation (OEF is defined as the ratio of the extracted oxygenation to arterial oxygenation). Rate of metabolism is calculated as the product of the assumed arterial oxygenation, OEF, and CBF.

An effective way to measure T_2_ values of blood in vivo is to apply T_2_ weighting “preparation modules,” which consist of ±90° hard pulses enclosing a train of refocusing pulses with different TEs, immediately before image acquisition.^71^ This approach has been applied to determine blood T_2_ of coronary veins,^72^ brain sagittal sinus,^73^, ^74^ and internal jugular veins.^75^, ^76^, ^77^ For abnormal blood composition such as sickle cell anemia, T_2_‐based oximetry may require disease‐specific calibrations.^77^, ^78^

The main challenge of this approach, however, is isolating signal solely from venous blood without contamination from tissue, CSF, or blood from other vascular compartments. Spin labeling methods provide a natural option to isolate vascular signal because the intrinsic subtraction can eliminate signal from unwanted voxel constituents.

T_2_‐Relaxation Under Spin Tagging (TRUST) was the first spin labeling technique to target venous blood signal.^73^, ^79^ TRUST modifies the pulsed ASL experiment by placing the inversion band above the imaging slab (instead of below) to invert venous spins flowing inferiorly. Control‐label subtraction yields high signal exclusive to medium‐to‐large size veins within the imaging slab. A T_2_ preparation module or FSE readout generates multiple echoes to fit for venous blood T_2_, ultimately yielding high‐SNR global oxygenation measurements in short scan times.

The Quantitative Imaging Of Extraction Of Oxygen And Tissue Consumption (QUIXOTIC) method expands on TRUST by employing velocity‐selective pulse trains to label blood accelerating from capillaries into the venous system. This allows T_2_ measurement of venous blood on a voxel‐by‐voxel basis, and generation of Y_v_, OEF, and CMRO2 maps. QUIXOTIC, however, is limited by low SNR and error introduced by CSF contamination.^80^ The Velocity Selective Excitation and Arterial Nulling (VSEAN) technique mitigates these limitations by applying a unique velocity‐selective excitation to acquire signal directly from slow‐moving venous spins, thereby improving SNR and reducing CSF contamination.^81^

#### 4.1.1 Suggestions

TRUST MRI uses a straightforward spin labeling approach to robustly measure global venous oxygenation and is recommended for most applications. It is easily translated to clinical and research settings due to high SNR, short imaging times, and simple data analysis methods. Furthermore, TRUST has been extensively tested and validated, including across multiple sites and in several disease states.^79^, ^82^, ^83^, ^84^, ^85^, ^86^ More advanced approaches such as QUIXOTIC or VSEAN allow voxel‐wise oxygenation measurements and reflect the next generation of spin labeling oxygenation methods. However, these are currently reserved for the expert user in specialized scenarios, given limited SNR and complex acquisition and analysis strategies.

### 4.2 MR fingerprinting ASL

A dynamic time series of images, in which the acquisition settings are varied in a pseudo‐random (but known) pattern, can be used to identify the underlying MR parameters of the tissue (e.g., its relaxation times).^87^, ^88^, ^89^, ^90^ The specific combination of tissue MR parameters at each voxel produces a unique dynamic MR signal for that specific acquisition, and this signal can be predicted in simulation. In MR fingerprinting, the parameter fits are carried out by identifying the signal from a precomputed database, or “dictionary,” of signals that matches the observed signal most closely. The entry that is most correlated with the observation corresponds to the appropriate combination of MR parameters.

The key features and advantages of the fingerprinting approach are that it produces joint parameter estimates from a given signal and is robust to spurious signals as long as their effect is not correlated with the parameter of interest. Joint parameter estimation of variables, like T_1_ and T_2_ relaxation, eliminates coregistration and other biases from separate measurements or assumptions. The dictionary matching process is generally very fast, but generating the dictionary is a computationally expensive process and can result in coarse granularity of the parameter estimates.

Fingerprinting is an appealing strategy in the context of quantitative ASL for several reasons. Primarily, ASL is intrinsically low SNR,^91^ and the robustness of fingerprinting to noise offers a major benefit. Second, quantification of ASL requires multiple parameters to be measured or assumed a priori. This can introduce biases into the measurement if assumed, or coregistration errors and additional scanning time if those additional parameters are measured separately. In contrast, ASL fingerprinting has been successfully implemented by collecting a single time series of PCASL prepared images in which the labeling duration varies according to a pseudo‐random, predetermined schedule, and the control/label condition of the PCASL preparation train is also randomized. A postlabeling delay is not necessary because the control PCASL periods serve as variable postlabeling delays for modeling as reduced flip angles are used to preserve some ASL signal from previous TRs. From this time series, multiple parameters can be estimated by matching the signal to a precomputed dictionary, usually T_1_ relaxation, perfusion, arterial blood volume, and bolus arrival time.

**FIGURE 5:**
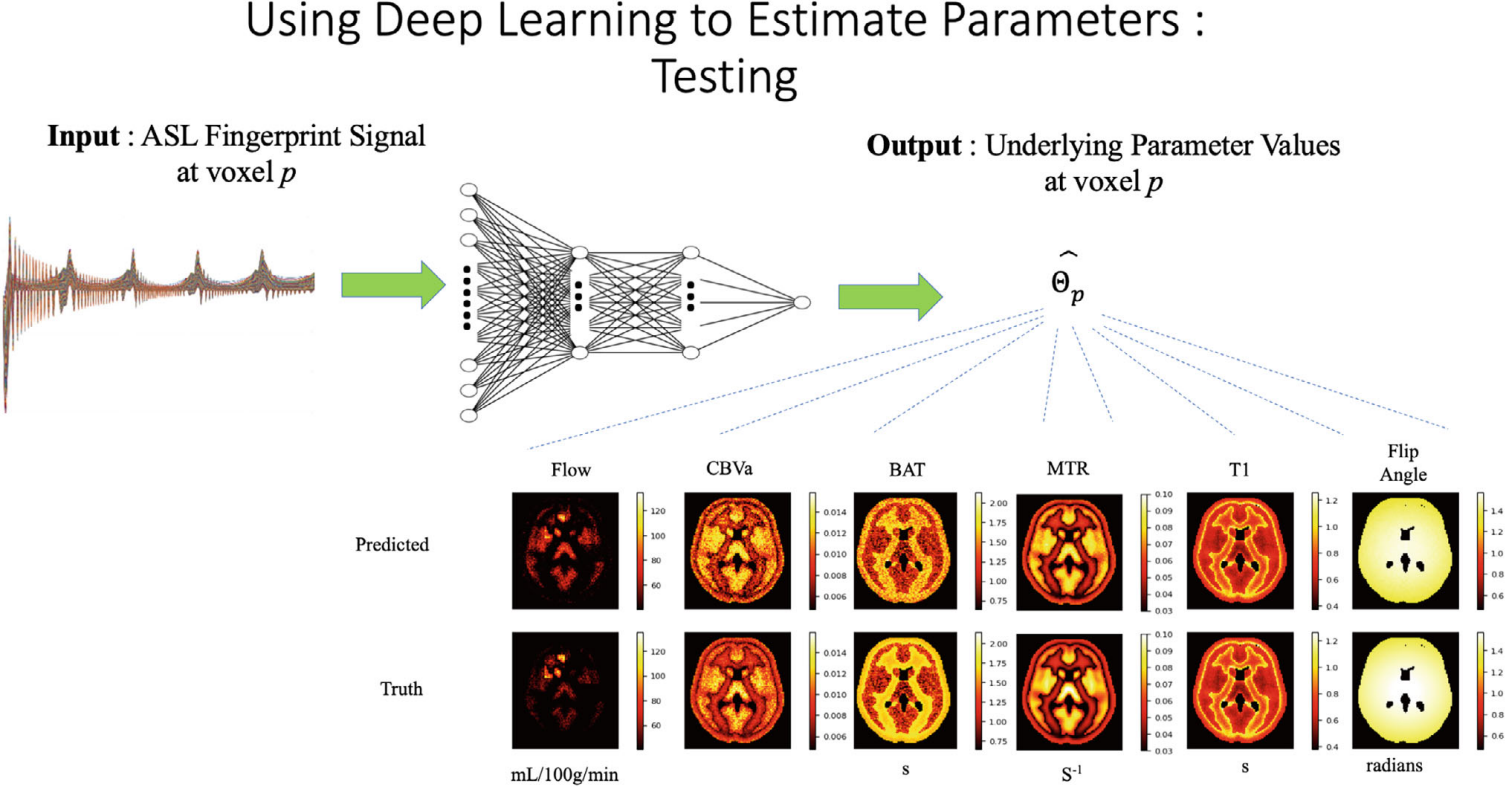
Example workflow of ASL fingerprinting using a neural network. Synthetic fingerprint signals are created from combinations of tissue parameters (perfusion, blood volume, bolus arrival time, magnetization transfer rate, T_1_, and flip angle in this case). These are used to train a set of neural networks that produce the parameter of interest as their output. Once trained, each network can estimate the underlying tissue parameters, given an experimental fingerprint time series. The diagram contains the results of simulations using synthetic parameter maps

In several studies, ASL fingerprinting with dictionary matching was able to estimate the hemodynamic parameters of interest, showing good agreement with more established ASL techniques.^92^, ^93^, ^94^, ^95^, ^96^ Recently, however, DL methods have been shown to be a powerful alternative to dictionary matching.^94^, ^96^ Whereas the data acquisition portion of the method remains the same, the parameter estimation portion can be accomplished more efficiently using neural network regression (Figure 5).

#### 4.2.1 Suggestions

ASL fingerprinting is a promising technique. Dictionary matching has been shown to be an effective way to estimate parameters, and neural network regression has been shown to offer clear advantages in terms of processing speed and granularity. However, ASL fingerprinting acquisition and processing methods are still evolving, so we refrain from making specific design suggestions at this point.

### 4.3 ASL angiography (ASL‐MRA)

ASL angiography (ASL‐MRA) has many advantages over conventional contrast‐enhanced MR/CT methods^97^: it allows vessel‐selective labeling (especially useful for assessing arterial supply to, for example, arteriovenous malformations/fistulas^98^, ^99^, ^100^, ^101^, ^102^, ^103^, ^104^) and has excellent flexibility in temporal and spatial resolution because the labeling and associated imaging readout can be repeated until the desired resolution is reached, unconstrained by the necessity to image the first passage of a contrast bolus.

To achieve high spatial resolution, however, the entire scan time is often used to acquire a large k‐space matrix without signal averaging. When vessel‐selective labeling is employed targeting multiple arteries, the total scan time can become very long. Therefore, the use of acceleration techniques should be considered: for example, undersampled golden‐angle stack‐of‐stars^105^ and 3D radial “koosh‐ball” acquisitions,^106^, ^107^ in conjunction with advanced image reconstruction techniques such as CS and k‐space weighted image contrast.^108^ Fortunately, ASL‐MRA is well‐suited for undersampled reconstruction because of its high sparsity in the image domain after subtraction, particularly when it is vessel‐selective.^109^

Both PASL and PCASL can be used for ASL‐MRA. PASL with a Look‐Locker readout has already proved its clinical usefulness in several studies^101^, ^104^, ^110^, ^111^ and is particularly good at visualizing the early inflow phase of the proximal arteries. However, vessel‐selective PASL has some difficulties (see below), which makes PCASL a preferred option for vessel‐selective MRA. PCASL can also be combined with subtraction techniques to visualize blood inflow.^112^, ^113^, ^114^

For static 3D‐MRA, in contrast, PCASL's long labeling duration is more advantageous for visualizing the whole arterial tree, and a hybrid of PCASL and PASL helps to minimize the signal loss in proximal vessels caused by fresh unlabeled blood flowing into the imaging volume.^107^, ^115^

Recently, velocity selective static 3D‐MRA^116^, ^117^, ^118^, ^119^ has also been demonstrated by utilizing Fourier transform‐based velocity selective saturation pulse trains, which set the flowing spins in the pass‐band and static spins in the saturation‐band before acquisition as a nonsubtractive method.

**FIGURE 6:**
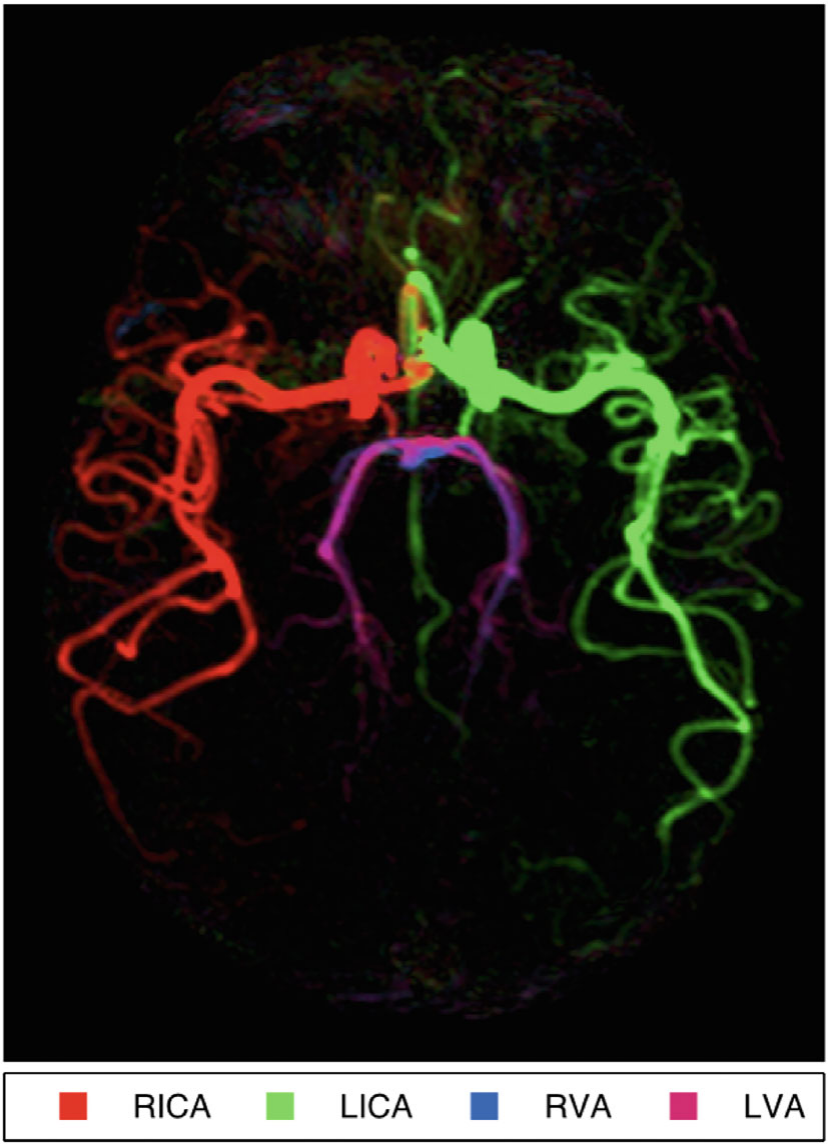
Example transverse maximum intensity projection frame from a vessel‐encoded dynamic angiography sequence acquired with a balanced steady‐state free precession readout (Okell et al., 2016). Color shows which proximal artery the blood signal originated from: the RICA/LICA or RVA/LVA RICA/LICA, right/left internal carotid artery; RVA/LVA, right/left vertebral artery.

The typical readout for ASL‐MRA is based on 3D gradient‐echo sequences. However, with a Look‐Locker readout, the repetitive excitation pulses can strongly attenuate the ASL signal when the flip angle is high. This can be mitigated through the use of a balanced steady‐state free precession readout (Figure 6) that recycles the transverse magnetization for the next excitation,^113^, ^120^ or the use of a segmented EPI readout to reduce the number of excitation pulses while making the interval between RF‐pulses longer.^97^, ^114^ However, off‐resonance effects can cause loss of vessel depiction with balanced steady‐state free precession,^113^ so high B_0_ homogeneity is required (e.g., using a small FOV or lower B_0_ field strength) and segmented EPI can suffer from ghosting due to strong pulsatile flow,^121^ typically at the M1 section of the middle cerebral artery, although this is reduced when using right–left phase‐encoding.

ASL‐MRA can be combined with perfusion imaging in a single sequence by sharing the labeling module, providing both macrovascular and microvascular information: besides CAPRIA^27^ (described earlier), time‐encoded PCASL can be combined with a segmented EPI 4D‐MRA readout, minimizing the number of excitation pulses required and preserving magnetization for a separate perfusion‐weighted readout.^122^

#### 4.3.1 Suggestions

For static 3D‐MRA, PCASL (ideally with PASL hybrid labeling) is recommended for visualizing the whole arterial tree. For 4D‐MRA, PASL with a Look‐Locker readout performs well for visualization of arterial blood as it flows into the brain. For vessel‐selective MRA, PCASL is the preferred option to avoid the difficulties associated with slab‐selective PASL. When employing PCASL, inflow subtraction should be considered to visualize the early inflow phase. Undersampled acquisitions in conjunction with advanced image reconstruction should be considered to minimize scan time. Readouts utilizing balanced steady‐state free precession or segmented EPI (with a factor of 3–7) help alleviate saturation of the ASL signal. However, in cases where B_0_ inhomogeneity or pulsatile ghosting are problematic, spoiled gradient‐echo sequences with low flip angles are recommended.

### 4.4 ASL functional MRI (fMRI)

Although hampered by its low SNR and acquisition speed, early work demonstrated that ASL offered several important advantages over blood oxygen level‐dependent (BOLD) fMRI. These include its quantitative nature and the temporal stability of the measurement—that is, it is not subject to 1/f noise that plagues BOLD fMRI.^40^, ^41^, ^123^, ^124^, ^125^, ^126^ These features make it more suitable for fMRI experimental paradigms that span longer periods of time (e.g., blocked designs with durations greater than a minute), such as applied in pharmacological fMRI or when studying conditions like sleep deprivation. For example, in an extreme case, images of the control and active conditions were taken 30 days apart and reliable activation maps of the motor cortex could still be obtained.^124^

Another advantageous feature of perfusion‐based (and blood volume‐based) fMRI is that CBF and cerebral blood volume changes are more specific to the parenchyma where the neural activity takes place, rather than the draining veins. This feature makes it particularly appealing for layer‐specific fMRI, where BOLD imaging is unable to differentiate activity between cortical layers.^127^, ^128^, ^129^, ^130^

ASL is also advantageous for fMRI in regions of high susceptibility‐induced static field inhomogeneities, such as the orbito‐frontal cortex, the amygdala, or the medial temporal lobe, where BOLD techniques are prone to signal loss because ASL does not depend on susceptibility contrast, and thus ASL images can be acquired using sequences with low T_2_* sensitivity.^131^ This feature of the technique makes it attractive for fMRI studies of spoken language because it is less sensitive than BOLD to speech‐related motion and susceptibility confounds.^132^, ^133^, ^134^

ASL‐based fMRI sequences typically avoid acquiring segmented readouts to ensure a sufficient temporal resolution. Besides the traditional 2D multislice EPI readout, 3D stack of spirals^135^ and 3D‐GRASE readouts^136^ are efficient approaches to collect all of k‐space after a single labeling/control period. An attractive acquisition strategy, using a pseudo golden‐angle stack‐of‐spirals 3D Rapid Acquisition with Relaxation Enhancement (RARE) readout and CS reconstruction, has been recently proposed that yields high spatial resolution time‐averaged CBF maps and low spatial resolution measurements of CBF fluctuations.^137^ More recently, velocity selective labeling pulses have been shown to allow faster sampling and improved sensitivity^138^ and could become more widely adopted for perfusion‐based fMRI.

ASL has also found some use for assessing resting‐state functional connectivity. Early on, it was shown that connectivity of the sensorimotor network could be detected with ASL by evaluating fluctuations in the CBF signal.^139^ Since then, several studies performed to identify resting state networks, applying different analysis methods, such as seed‐based connectivity,^140^, ^141^, ^142^ independent component analysis^143^, ^144^, ^145^, ^146^, ^147^ and whole‐brain voxel level connectivity,^143^, ^148^ have found similar brain networks as those observed in resting state BOLD studies. As in the case of task activation studies, resting‐state functional connectivity measured with ASL can potentially provide better localization of resting state networks than BOLD, despite the lower spatial resolution of the ASL images. The lower temporal resolution of ASL is not so much of a disadvantage because resting state connectivity is based on the correlation of low frequency signal fluctuations.

#### 4.4.1 Suggestions

ASL‐based fMRI can be achieved by combining a labeling scheme with a fast volumetric readout, such as a stack of spirals, combined with parallel imaging acceleration schemes. Background suppression and time‐series denoising techniques (see previous sections) can be extremely helpful for detecting activation. Velocity selective ASL has been shown to be advantageous because it allows faster sampling, given the negligible bolus arrival delays. ASL‐based techniques hold great promise in layer‐specific fMRI.

## 5 VESSEL‐SELECTIVE ASL

Often the total amount of blood perfusing a particular region of tissue is the main parameter of interest, but in some situations it is also desirable to know which artery the blood signal originated in. One of the great advantages of ASL over other perfusion imaging modalities (e.g., positron emission tomography, single photon emission computed tomography) is the ability to image the perfusion territory of a specific artery. The perfusion territories of the brain‐feeding arteries demonstrate a wide variability due to anatomical variations in the cerebral vasculature and hemodynamic changes caused by cerebrovascular disease.^149^ Clinical applications of territorial perfusion imaging include assessment of collateral flow patterns in steno‐occlusive disease and identifying the blood supply to ischemic lesions, arteriovenous malformations, or tumors.^150^

### 5.1 Slab‐selective single artery labeling

Some of the original techniques for vessel‐selectivity restricted the spatial region over which an ASL inversion pulse acted, thereby only labeling a single vessel at a time. The most common approach is to use a conventional slab‐selective inversion pulse but to angle it in such a way as to only cover the artery of interest.^151^, ^152^, ^153^ Efficient postlabeling saturation must then be used to remove any effect of the angled labeling pulses on tissue magnetisation within the imaging region. However, orientating the slab to cover only the artery of interest, which is often tortuous, is challenging. In addition, if only a limited vessel segment can be covered, then the bolus of labeled blood created is relatively small, the SNR of the resulting images is impaired, and perfusion quantification is challenging.

### 5.2 Superselective methods

Vessel‐selective labeling based on (P)CASL avoids some of the drawbacks of the slab‐selective PASL‐based methods by using a secondary gradient perpendicular to the main labeling gradient axis. If the gradient is rotated dynamically during the labeling period instead of applying this gradient in a continuous fashion, one can achieve a small labeling region. Early vessel selective work using CASL essentially created a labeling plane that was not perpendicular to the flow direction and rotated about a target artery such that only the spins flowing through that artery would experience the adiabatic inversion that underlies CASL.^154^, ^155^

**FIGURE 7:**
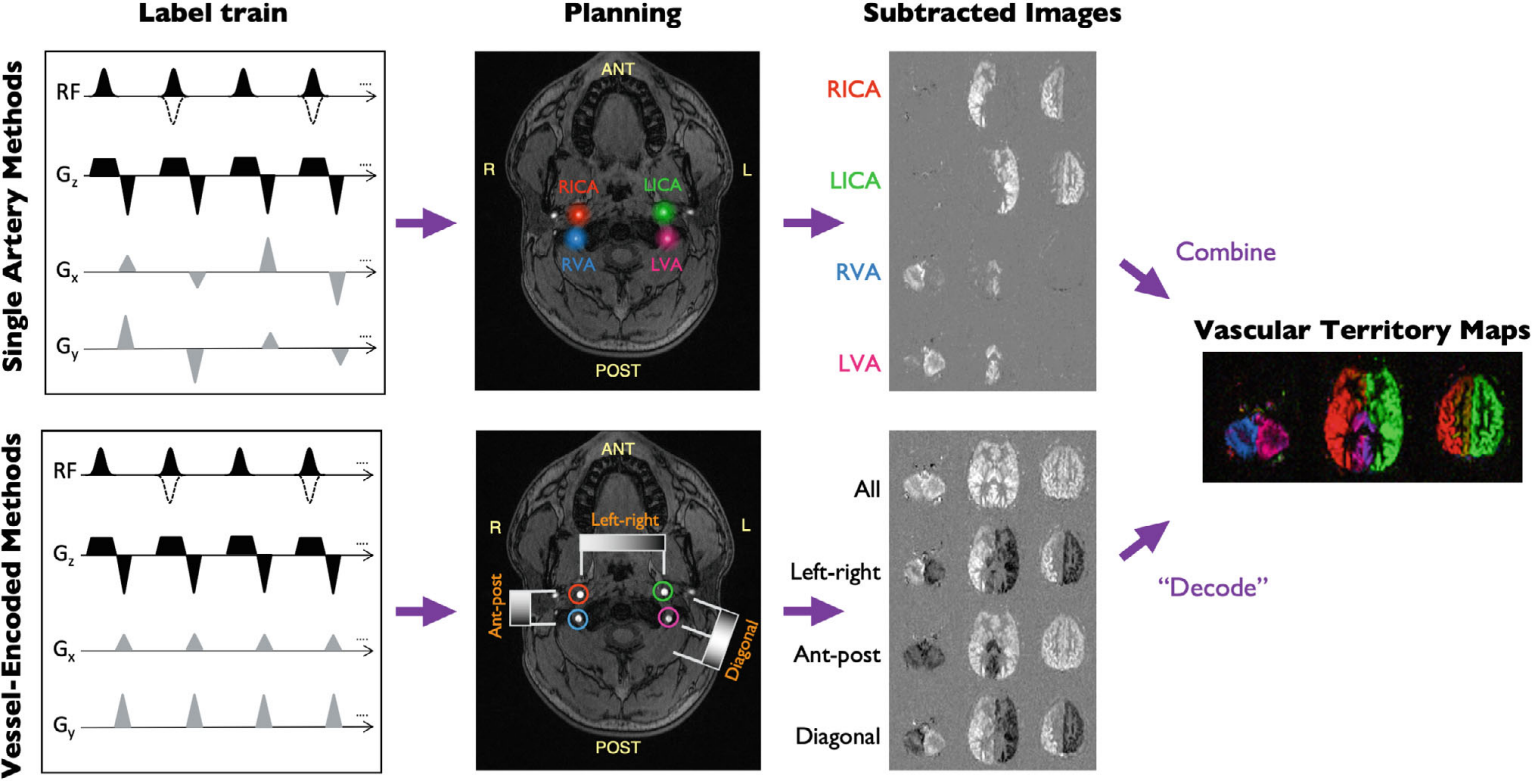
Vessel‐selective PCASL methods: The pulse sequence diagrams (left) of superselective (top) and vessel‐encoded (bottom) PCASL are very similar. For superselective labeling, the in‐plane gradient blips (Gx, Gy) are rotated every RF pulse in a continuous or pseudo‐random fashion, generating a single labeling “spot” (middle). Dotted RF lines represent the control condition. For vessel‐encoding, the gradient blips are applied in a consistent direction, creating bands of label and control conditions across the labeling plane that are are varied across a number of encoding cycles. For superselective labeling, each artery of interest is labeled separately (middle) and then combined (right). For vessel‐encoding, each encoding cycle generates images with different combinations of arteries in (ideally) label or control conditions, which are combined in postprocessing to identify the signal arising from each artery. Color is used here to represent the origin of the blood signal (red = right internal carotid; green = left internal carotid; blue = right vertebral; magenta = left vertebral). PCASL, pseudo‐continuous arterial spin labeling.

A similar idea can be applied to PCASL methods by inserting in‐plane gradient pulses between the individual RF labeling subpulses that make up a balanced PCASL labeling train (see Figure 7). The effect is a phase distribution of the spins determined by their location along the in‐plane gradient direction. Matching the phase of the individual pulses in the labeling train to the phase of the spins at a specific vessel location allows the creation of a “labeling stripe” that tags spins flowing through that location by adiabatic inversion similar to nonselective PCASL. The periodic nature of phase accrual means that if the in‐plane gradient pulses were the same each time, these conditions would be met at a number of stripes within the labeling plane. In superselective PCASL, the in‐plane gradient is rotated at varying increments between RF pulses in the PCASL train (in a continuous or pseudo‐random fashion), and the RF phase adjusted such that only the spins flowing through 1 location in the plane will experience the adiabatic inversion process.^156^, ^157^

The amplitude of the in‐plane gradient blips determines the effective “labeling spot” size and must be chosen as a compromise between labeling efficiency/insensitivity to motion (larger spot size) and the potential for labeling other nearby arteries (smaller spot size). Moreover, the labeling plane needs to be oriented approximately perpendicular to the artery, intersecting at a straight part of the artery and without intersecting the tissue in which the relevant imaging is performed.

Superselective PCASL has already shown some promising results in patients with a range of cerebrovascular diseases, including steno‐occlusive disease and arteriovenous malformation.^150^, ^158^ Recent work on correcting for off‐resonance effects and pulsatility is likely to further improve robustness.^33^

### 5.3 Vessel‐encoding

Given a limited scan time, labeling methods with higher time efficiency are preferred, that is, methods that can label several feeding arteries simultaneously either by pulsed^159^ or (pseudo‐) continuous labeling methods.^160^, ^161^ In this type of approach, perfusion images are acquired in a few “encoding” steps. As described earlier (see Figure 7), including an additional gradient blip within the PCASL labeling plane in a consistent direction, along with associated RF phase modulations, creates spatial labeling bands within the plane without labeling the blood in other regions. The encoding of arteries is achieved by labeling different subregions of the labeling plane over a series of readouts. For PASL‐based approaches, this involves positioning the labeling slab to cover more than 1 artery at a time, although the difficulties in positioning this slab to cover tortuous arteries still remain, so PCASL‐based approaches are generally preferred.

In each of several readouts, the feeding arteries are labeled and encoded differently, for example, inverted (label) and unperturbed (control) arterial magnetization are encoded as −1 and 1, respectively. With the tissue signal always encoded as 1, an encoding matrix can be constructed to describe the signals acquired for all the encoding steps at the imaging slices,^160^ for example,

$$\left[\begin{array}{c} y_{1}\\ y_{2}\\ y_{3}\\ y_{4} \end{array}\right]=\left[\begin{array}{cccc} -1 & 1 & -1 & 1\\ 1 & -1 & 1 & -1\\ -1 & -1 & 1 & 1\\ 1 & 1 & 1 & 1 \end{array}\right]\times \left[\begin{array}{c} L\\ R\\ B\\ T \end{array}\right]$$

where the measured signal vector, $y={\left[y_{1}\;y_{2}\;y_{3}\;y_{4}\;\right]}^{T}$ and $y_{i}$ is the signal acquired in step i; the signal source vector $x={\left[L\;R\;B\;T\;\right]}^{T}$; and L, R, B, and T are the signals from the left carotid, right carotid, basilar arteries, and brain tissue, respectively. The observed signals (y) are a linear combination of the contributions (x), mixed by the encoding matrix, A, made of 1 and − 1. The contribution from each feeding artery can then be calculated by $x=A^{-1}y$, where $A^{-1}$ is the inverse or pseudo‐inverse of the encoding matrix A.

Using columns from a Hadamard encoding matrix (with elements of 1 or − 1) to construct the encoding matrix,^160^ such as the one shown above, maximizes encoding and SNR efficiency.^159^ This leads to vessel‐encoded ASL, sometimes being referred to as *Hadamard‐encoded* ASL, although this should not be confused with time‐encoded methods, which also use Hadamard encoding.^162^ To distinguish N vascular territory regions, the SNR for each feeding artery using Hadamard encoding is improved by a factor of $\sqrt{N}$ compared to labeling each feeding vessel individually,^159^ given the same total acquisition time.

Due to variation in the geometry of the feeding arteries and scanner hardware limitations, Hadamard encoding schemes may not always be feasible, or the planning/calculation process could be slow, although some automated methods to optimize the encodings have been proposed.^163^, ^164^, ^165^ Optimization of the labeling parameters can also improve the separation of arteries selected to be in label or control conditions.^161^, ^166^

Due to field inhomogeneities such as B_1_ variation or off‐resonance at the labeling sites, the actual labeling status of the feeding arteries may deviate from the designed values (e.g., encoded as 0 if the signal is saturated) and should be estimated from the data to accurately decode the vascular territory information.^160^ This can be done by estimating the encoded labeling efficiency of the ASL signal in each perfusion territory by k‐means clustering and linear analysis,^160^ or by using Bayesian inference framework with improved accuracy.^167^

Some applications of vessel‐encoded ASL include detecting/assessing collaterals,^168^ or producing vessel‐encoded angiograms^169^ that can be used to assess the blood supply to arteriovenous malformations.^170^

### 5.4 Suggestions

PCASL is the recommended method for vessel‐selective ASL. When choosing between the vessel‐encoded and superselective labeling schemes, the purpose of the scan should guide the decision: when there is need to have insight into all (or the main) flow territories, vessel‐encoded labeling using a Hadamard scheme is the most efficient method and will yield the highest SNR. However, when there is specific interest in the flow territory of a single or a few arteries, especially in cases where these arteries are located intracranially or are part of an unusual vascular anatomy, superselective labeling is the method of choice: it allows the labeling plane to be optimally positioned for each artery and is perhaps the simplest to implement. However, in both methods, imperfect labeling efficiency must be accounted for when trying to quantify CBF or mixed perfusion fractions.

## 6 DEEP LEARNING IN ASL

Machine learning (ML) applications are on a steep rise in the domain of medical imaging. Special attention should be given to deep convolutional neural networks, which have shown excellent performance in medical image analysis tasks.^171^ These methods are further supported by growing initiatives for public data sharing, which enables building of large multi‐center datasets that are key in the effort to reliably train and validate a machine‐learning model. Historically, multi‐site ASL data sets have been notoriously difficult to combine due to intervendor implementation differences and a lack of protocol standardization; the previous consensus paper^3^ has helped to address these issues, and current efforts to standardize parameter notation as part of the new ASL Brain Imaging Data Structure (BIDS) extension^172^ and the Open Science Initiative for Perfusion Imaging also aim to improve harmonization.

This is a rapidly developing area, and we expect many new innovations to occur in the coming years. So far, 4 main types of tasks are typically solved using ML methods: parameter estimation, image denoising (described above), predicting images with different contrast, and directly predicting diagnosis or disease severity.

### 6.1 ASL quantification

DL provides a powerful way for solving complex nonlinear inverse problems, such as the one posed by ASL, particularly in the fingerprinting application (described above). In the case of ASL fingerprinting, neural network regression can be used to estimate multiple parameters independently, 1 at a time, without assuming the value of the other parameters.

The general strategy is to generate a database of synthetic signals based on a physics‐based model, the pulse sequence parameters (e.g., labeling duration schedule, PLD, TR), and many parameter combinations. This database of signals is then used to train a set of neural networks to output the desired parameters. Once trained, each of the networks will take the observed signal as input and yield a parameter estimate as its output. Alternatively, experimental ASL data from a high‐quality data set in which the underlying parameters were known a priori can be used to train the neural networks instead of using purely synthetic data from Bloch simulations.^96^

Training the neural networks requires a large database of signals, which is computationally expensive to synthesize and store. However, the network needs only to be trained once. After training, computation of the output (i.e., the parameter estimates) is extremely fast. This approach offers an important advantage over dictionary learning: it allows for much finer granularity of the parameter estimates, whereas the dictionary entries are computed on a coarser grid of parameter values because the size of the dictionary grows exponentially with the grid size and the number of parameters (dimensions) that one wishes to estimate.

In terms of ASL, this strategy has been demonstrated to estimate hemodynamic parameters from ASL fingerprints quite effectively,^94^, ^96^ although a fingerprint's sensitivity to perfusion and other hemodynamic parameters can be limited in some cases.^94^ Optimizing the fingerprint readout schedule to maximize the sensitivity to perfusion (using an objective metric of sensitivity, such as the Cramer‐Rao bound) is crucial to obtaining reliable estimates. As a result, perfusion, arterial transit time, and arterial blood volume can be estimated reliably in addition to T_1_ relaxation time and the effective flip angle, giving good agreement with standard measurements.^94^, ^96^

### 6.2 Machine learning and ASL for diagnosis

ML and DL give us the means to study regional and voxel‐wise patterns of pathological perfusion changes in more detail than a simple evaluation at specific pathology‐related regions. Two distinct approaches are generally used for ASL: (i) evaluation of regional mean CBF in anatomical regions based on atlases and then working in the vector space defined by these regions to, for example, separate healthy controls from patients with a major depressive disorder^173^; and (ii) process the full voxel‐wise CBF maps either using DL based on neural networks, or using a feature space reductions methods (such as PCA) and traditional ML algorithms (such as a support vector machine). Although DL‐based methods can achieve higher performance and are not bound to predefined anatomical regions, such methods have numerous shortcomings: Much larger datasets are needed for training, and they suffer from interpretability issues, can cue on nonperfusion‐based artifacts such as motion, and are computationally more demanding. The major hurdle is, however, the sensitivity of the ASL protocol: variations in acquisition parameters (commonly present in ASL) can render a well‐performing machine learning method useless on another acquisition protocol.

Despite the first examples of ML/DL applications emerging, they are still pilot studies conducted on a limited number of patients from a single cohort without an external validation and are thus far from wider adoption in clinical research. Standardizing image processing to decrease the between‐center differences in data^174^ is a way to gather larger datasets, necessary for both the ML and DL training.

### 6.3 Suggestions

Whereas ML offers great promise, this field is still evolving. We anticipate the continued development and validation of these techniques for ASL, particularly those that are robust to differences between sites, scanners, and acquisition protocols.

## 7 ULTRAHIGH FIELD: ASL AT 7 T

**FIGURE 8:**
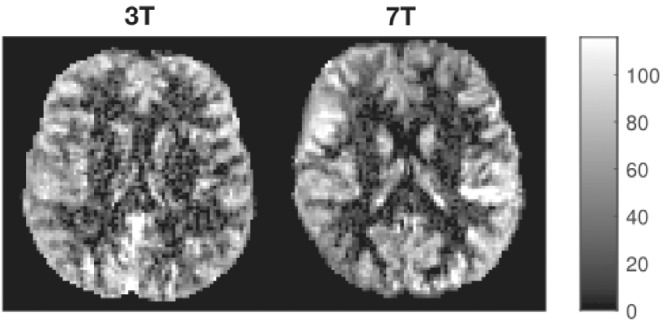
Example PCASL CBF maps (in mL/100 g/min) generated in the same subject using the same protocol at 3 T and 7 T. At this resolution (2 × 2 × 4 mm) the 3 T data is relatively noisy, but the SNR increase at 7 T gives a considerable improvement in image quality. However, in order to achieve reasonable quality perfusion images at 7 T, the labeling plane had to be positioned within the brain to avoid severe B_0_ and B_1_ inhomogeneities, meaning whole brain coverage was not possible. In addition, the label duration had to be kept short (1400 ms), and it was only possible to use presaturation for background suppression because additional inversion pulses would have exceeded SAR limits. Other imaging parameters: PLD = 2000 ms, TR = 4000 ms, readout scheme = 2D multi‐slice EPI, number of slices = 10, TE = 13 ms, parallel imaging (GRAPPA) factor = 2, scan time = 5 min. SAR, specific absorption rate; T, Tesla.

ASL should benefit at higher B_0_ field strengths from both the intrinsic SNR increase and the longer T_1_ relaxation time of blood. This large boost in SNR could be traded off for shorter scan times, higher spatial resolution, and/or increased sensitivity to low levels of perfusion (e.g., in the WM of the brain). The potential for improved SNR can be seen in PCASL images collected at 3 tesla (T) and 7 T in Figure 8. PCASL images collected at 3 T and 7 T can be seen in Figure 8. However, a number of technical challenges have prevented the widespread use of ASL at ultrahigh field (UHF).^175^ These include: (i) increased main field (B_0_) inhomogeneity; (ii) increased transmit RF (B_1_^+^) inhomogeneity, often with limited coverage; (iii) increased power deposition; (iv) more rapid T_2_/T_2_* decay; and (v) increased physiological noise.

Much of the early work on UHF ASL made use of a pulsed ASL preparation and imaging of only a limited region of the brain.^176^, ^177^, ^178^ More recent work utilizing optimized PASL inversion pulses^179^ as well as dielectric pads and simultaneous multi‐slice EPI,^6^ has demonstrated improved labeling efficiency, brain coverage, and temporal resolution. Such techniques show great promise, particularly for high spatial resolution functional imaging,^129^, ^180^ such as laminar fMRI, without the confound of draining veins that can bias conventional BOLD‐based methods. Although promising, the main limitation of PASL at UHF is that labeling can only occur within a spatial region defined by the transmit RF coil: at 7 T, this is typically a head‐only transmit coil, unlike the body coils used at lower field strengths. Therefore, there is a tradeoff between brain coverage and the remaining region within the head coil that is available for generating the bolus of labeled blood, which directly impacts the achievable SNR.

PCASL has the potential to overcome this obstacle because only the thin labeling plane must be located within the sensitive region of the transmit coil: generation of long boluses of labeled blood should therefore still be possible while maintaining whole‐brain coverage. However, PCASL is also particularly sensitive to all of the technical issues mentioned above, so much of the work in this area has focused on tackling these. B_0_ inhomogeneity can be mitigated using a prescan to estimate field offsets at each vessel location, which can then be corrected using transverse gradient blips between PCASL pulses^181^ or phase correction schemes.^182^ Reduced B_1_^+^ amplitude in the labeling region can be partially compensated using high‐permittivity pads,^183^ whereas transmit homogeneity at the labeled vessel locations can be improved using B_1_^+^ shimming.^184^, ^185^ Both approaches also help to improve transmit efficiency, reducing power deposition, particularly when variable rate selective excitation is applied,^184^, ^186^ although this often appears to remain a limiting factor. Fast Low Angle Shot‐based readouts show promise for limiting the impact of short T_2_ decay at 7 T and are potentially more robust to physiological fluctuations.^187^, ^188^

Despite these advances, it has proven difficult to realize the full theoretical potential of ASL at UHF. Future work to further reduce power deposition, allowing optimal labeling durations and background suppression to be achieved and perhaps utilizing full parallel transmission capabilities, is likely to help push this field forward in the future.

### 7.1 Suggestions

UHF PASL using appropriately optimized inversion pulses could be considered when very high spatial resolution is required, particularly for layer‐specific functional imaging, although this becomes more challenging in inferior brain regions. Whereas UHF PCASL shows great promise, technical challenges such as B1 inhomogeneity and power deposition have thus far hindered its implementation, so further work in this area is encouraged to allow optimal labeling durations and background suppression to be achieved, perhaps utilizing full parallel transmission capabilities.
